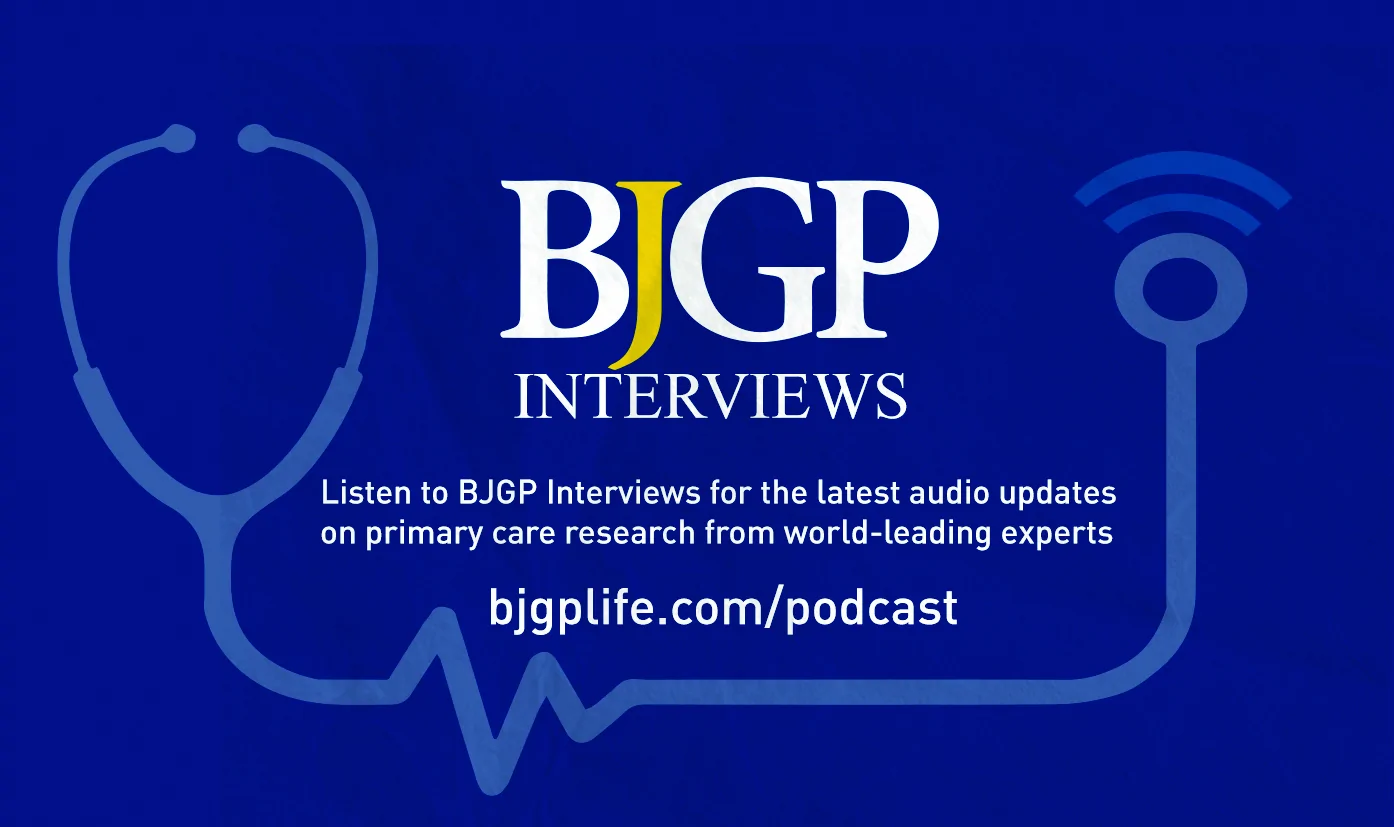# Scottish Referral Guidelines for Suspected Cancer (2025): key clinical changes

**DOI:** 10.3399/BJGP.2026.0194

**Published:** 2026-09-01

**Authors:** Charlotte Williamson, Lorna Porteous, Phil Hodkinson, Sowmiya Moorthie, Samantha Harrison

**Affiliations:** 1 Cancer Research UK, London, UK; 2 NHS Lothian, Edinburgh, UK; 3 Consultant Respiratory Physician, NHS Ayrshire and Arran, East Ayrshire, UK; 4 Clinical Lead for Earlier Cancer Diagnosis, Centre for Sustainable Delivery, NHS Scotland, Scotland, UK

In Scotland, the most common route to cancer diagnosis is via general practice.^1^ Timely recognition of suspected cancers in primary care, appropriate testing, and referral along urgent cancer pathways can improve patient outcomes through earlier diagnosis.^2^

Recognising suspected cancers in primary care is challenging. There are limited tests available to support assessment, and most symptoms are not strongly predictive of cancer. Cancer referral guidelines support health professionals in their practice and ensure a consistent approach to cancer investigation. The latest version of the Scottish Referral Guidelines for Suspected Cancer (SRGs) were published in August 2025.^3^

The Scottish Government commissioned the Centre for Sustainable Delivery (CfSD) to conduct a full clinical review of the SRGs. Cancer Research UK (CRUK) and Healthcare Improvement Scotland provided evidence and insight to support the process.

CRUK conducted 16 rapid literature reviews across all cancer sites included in the guidelines, and for non-specific symptoms. The findings were distributed to expert stakeholders (including primary and secondary care, third sector, PPI representatives) and discussed at peer review sessions, supporting initial decisions about guideline changes. A final consultation phase maximised stakeholder feedback and reached consensus on guideline changes.

## How have the guidelines changed?

General guideline changes included improving clarity, ensuring consistent terminology, and including additional information to support health professionals in their decision making around wider considerations that may impact their practice. For example, site-specific information was included on inequalities that certain patient groups may experience in access, experience, and outcomes, and safety-netting guidance. There are several themes that demonstrate how the guidelines evolved to align with the changing evidence base, as outlined below.

## Increasing risk stratification where evidence supports it

There is a growing evidence base demonstrating how different combinations of clinical data can provide more detailed information about cancer risk.

Symptoms that warrant a chest X-ray (CXR), for instance, are now stratified by smoking status in the updated lung guidelines. If somebody has never smoked, they require two symptoms in combination to warrant a CXR, whereas those who have ever smoked or currently smoke only require one. This aligns with the evidence base which suggests that the risk of lung cancer in those who smoked is higher than those who have never smoked.^4^ Additionally, the criteria for those who warrant an urgent suspicion of cancer (USC) referral following a normal CXR have been refined. This is based on evidence highlighted in CRUK’s evidence reviews, demonstrating certain symptom combinations have a significant lung cancer risk^5^ despite a negative CXR; for example, weight loss and thrombocytosis.

Risk stratification helps identify patients at highest cancer risk while managing low-risk patients in primary care. For example, the same presentation may lead to reassurance and safety netting in someone deemed low risk, but prompt USC investigation or referral in those at higher risk. This can provide more personalised care, but it also adds complexity to guidelines. It is imperative that health professionals are familiar with the recommendations. Figure 1 outlines the key clinical changes across the guidelines.

**Figure 1.**
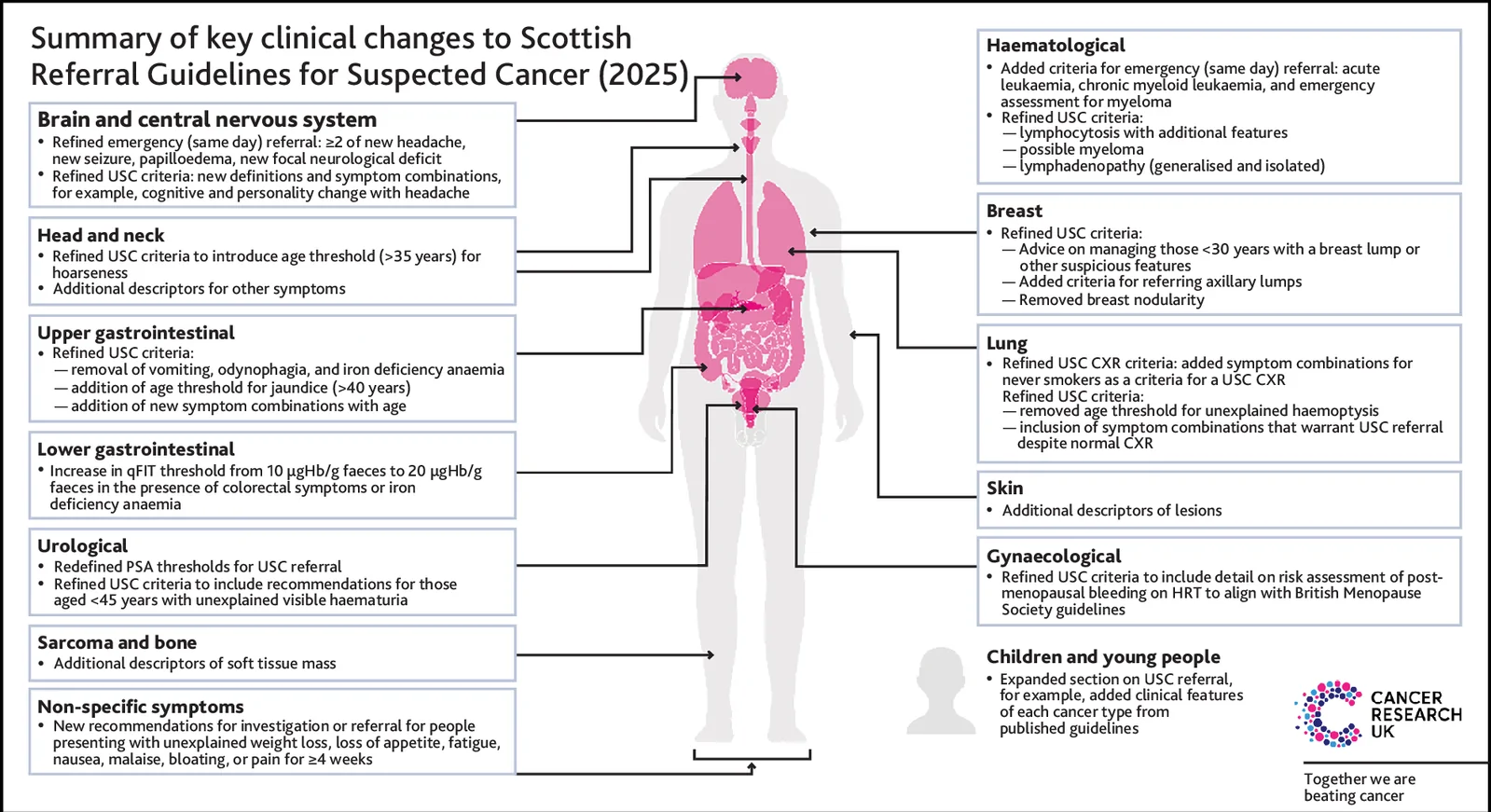
Summary of key clinical changes to Scottish Referral Guidelines for Suspected Cancer (2025). Copyright Cancer Research UK. Used with permission. CXR = chest X-ray. HRT = hormone replacement therapy. PSA = prostate-specific antigen. qFIT = quantitative faecal immunochemical test. USC = urgent suspicion of cancer.

## Aligning with other national guidelines

The SRGs refer to other national pathways and UK-wide guidelines, to ensure there is alignment, making referral decisions easier to navigate.

For example, the lower gastrointestinal guideline is aligned with the national quantitative faecal immunohistochemical testing (qFIT) consensus document.^6^ The qFIT threshold for USC referral has increased from ≥10 to ≥20 µgHb/g faeces and provides detailed advice on how to manage those with a FIT <20 µgHb/g faeces.

Additionally, the endometrial guideline aligns with the British Menopause Society’s management of unscheduled bleeding on hormone replacement therapy (HRT) guidelines.^7^ The recommendations outline best-practice management for people presenting with post-menopausal bleeding, based on risk factors such as HRT status.

## Considering non-specific symptoms

Over half of patients diagnosed with cancer present with non-specific symptoms (NSS).^8,9^ Determining onward management of this patient group poses a challenge for primary care. Patients who present with NSS often experience prolonged primary care and diagnostic intervals, later-stage diagnosis, and diagnosis via emergency presentation.^10^

Roll-out of Rapid Cancer Diagnostic Services (RCDS) (or direct-access CT where RCDS is not available) in Scotland has provided a route for GPs to refer patients presenting with NSS on to a managed pathway. To align with these pathways, SRGs include a new set of recommendations on managing NSS, which is a unique characteristic of this guideline compared with other national referral guidelines. SRGs also note the importance of a GP’s ‘gut feeling’ as a diagnostic aid that has been shown to be predictive of cancer^11^ and can be used to support rationale when referring to RCDS.

The SRG update is a great example of how to develop high-quality, evidence-based national guidelines by triangulating existing guidance, evidence, and expert clinical insight. CRUK has developed a host of resources that support health professionals’ guideline use, which can be found at CRUK’s health professionals’ hub.^12^

**Figure:**